# Supplementary material for: Establishment of Novel Gastric Cancer Patient-Derived Xenografts and Cell Lines: Pathological Comparison between Primary Tumor, Patient-Derived, and Cell-Line Derived Xenografts
Source: Cells. 2019 Jun 14;8(6):585. doi: 10.3390/cells8060585 (PMC6627523; doi:10.3390/cells8060585)
Supplement: Supplementary file 1 [file cells-08-00585-s001.pdf]

Table S1 Average number of weeks of tumor growth for xenograft expansion until tumor harvesting

Histology of primary tumor	n	Generation of xenograft: mean ± SD (min–max)				
		1st	2nd	3rd	4th	5th
DA	27	17.4 ± 14.8 (4.3–58.1)	10.4 ± 7.7 (3.1–35.1)	7.4 ± 3.8 (2.9–16.0)	8.8 ± 7.0 (2.1–35.0)	7.2 ± 3.8 (2.4–19.7)
PDA	8	16.8 ± 15.6 (8.4–57.9)	20.2 ± 18.0 (4.0–59.7)	14.6 ± 11.5 (5.0–42.0)	13.2 ± 8.8 (5.9–33.0)	9.5 ± 5.5 (4.1–21.0)

DA: Differentiated adenocarcinoma, PDA: Poorly differentiated adenocarcinoma

Table S2 Pathological factors related to PDX establishment (stratified by type of immunodeficiency mouse model)

Mouse	pT/N/M		PDX		p-value
			Fail	Success	
NOG	pT	1/2	38	7	0.62
		3/4	95	14	
	pN	0	47	2	0.02
		1<	86	19	
	pM	0	128	21	1.00
		1	5	0	
NGS	pT	1/2	0	0	-
		3/4	5	0	
	pN	0	1	0	-
		1<	4	0	
	pM	0	5	0	-
		1	0	0	
SCID	pT	1/2	9	2	0.61
		3/4	25	3	
	pN	0	10	1	1.00
		1<	24	4	
	pM	0	34	5	-
		1	0	0	

Table S3 Pathological factors related to PDX establishment (adenocarcinoma cases only, stratified by type of histological differentiation grade)

Histology of primary tumor	pT/N/M		PDX		p-value
			Fail	Success	
DA	pT	0	30	6	0.10
		1	57	12	
	pN	0	34	2	0.03
		1	53	16	
	pM	0	86	18	1.00
		1	1	0	
PDA	pT	1/2	15	3	0.20
		1	66	5	
	pN	0	23	1	0.44
		1	58	7	
	pM	0	77	8	1.00
		1	4	0	

DA: Differentiated adenocarcinoma, PDA: Poorly differentiated adenocarcinoma

Table S4 PDX establishment success and tumor location

	PDX		
	Failed	Success	Success rate
EGJ	8	2	20.0%
U	41	4	8.9%
M	44	5	10.2%
L	79	14	15.1%
N/A	0	1	0.0%

EGJ: Esophagogastric-junction, U: Upper, M: Middle, L: Lower, N/A: Not available
